# Nasal Myiasis Associated With *E. coli* Bacteremia, Spinal Osteomyelitis, and Epidural Abscess: A Case Report

**DOI:** 10.1155/crdi/4888769

**Published:** 2026-07-27

**Authors:** Faith Miller, Jesse McLean, Giulio DiDiodato

**Affiliations:** ^1^ Department of Critical Care Medicine, Royal Victoria Regional Health Centre, Barrie, Ontario, Canada; ^2^ Department of Family and Community Medicine, University of Toronto, Toronto, Ontario, Canada, utoronto.ca

**Keywords:** *E. coli* bacteremia, epidural abscess, nasal myiasis, spinal osteomyelitis

## Abstract

**Introduction:**

Nasal myiasis is a rare parasitic infestation that can predispose to secondary bacterial infection, though progression to systemic complications is uncommon. Cases in nonendemic regions are rare and may present diagnostic challenges.

**Case Presentation:**

We report a case of community‐acquired nasal myiasis in a 65‐year‐old woman presenting with an altered level of consciousness, found to have *E. coli* bacteremia and subsequent spinal osteomyelitis complicated by an epidural phlegmon requiring surgical decompression. Larvae were directly visualized within the anterior nasal cavity, with no evidence of deeper airway involvement or additional sites of infestation. Despite extensive investigation, including abdominal and pelvic imaging, no alternative infectious source was identified. Intraoperative cultures were negative following approximately 16 days of antimicrobial therapy.

**Discussion:**

Although a direct microbiological link between the nasal infestation and bacteremia could not be established, the clinical findings support a plausible secondary bacterial invasion facilitated by mucosal disruption.

**Conclusion:**

This case highlights the potential for severe systemic complications arising from nasal myiasis and underscores the importance of early recognition and thorough evaluation of atypical sources of bacteremia.

## 1. Introduction

Nasal myiasis is a rare parasitic infestation caused by fly larvae, most often seen in tropical and subtropical regions [1, 2]. In temperate, nonendemic regions such as Canada, cases are uncommon and typically nosocomial in origin [3, 4]. Reports of community‐acquired nasal myiasis, especially in returning travelers, remain scarce and can pose diagnostic challenges when presenting with altered mental status or comorbidities.

Although myiasis can predispose individuals to secondary bacterial infections, progression to systemic sequelae is highly unusual [5, 6]. Here, we describe a unique case of nasal myiasis in a 65‐year‐old woman, associated with *E. coli* bacteremia and severe spinal infection requiring surgical decompression. This case underscores the potential for rapid systemic deterioration and the importance of early recognition.

## 2. Case Presentation

A 65‐year‐old woman was admitted to the hospital and intubated for an altered level of consciousness (Day 0). The patient had a history of hypertension and major depressive disorder. She was a lifelong nonsmoker, self‐reported alcohol use estimated at less than 7 alcoholic equivalents per week, and denied recreational drug use. There was no documented history of diabetes mellitus, chronic liver disease, immunosuppression, or other conditions associated with increased risk of myiasis. She had recently returned from trips to Mexico (Day ‐12) and the west coast of Canada (Day ‐7). She was last seen awake by her partner at 2:00 a.m. (Day 0) taking recently prescribed baclofen for neck and back pain (Day ‐5); she had taken 50 mg of baclofen within 24 h prior to admission.

Urine toxicological screen was negative for nonprescribed medications. Ethanol level was negative. She was hemodynamically stable and afebrile. Computed tomography brain imaging and chest X‐ray were nondiagnostic. Initial laboratory investigations demonstrated a white blood cell count of 7.94 × 10^9^/L, hemoglobin of 98 g/L, and platelet count of 39 × 10^9^/L. Serum biochemistry revealed an elevated creatinine of 281 μmol/L. Blood cultures obtained on admission grew *E*. *coli* in both aerobic and anaerobic bottles, with time to detection of 15 h 53 min and 21 h 24 min, respectively. Infectious diseases work‐up included blood cultures and lumbar puncture (Day 0), the latter initially unsuccessful. Empiric treatment with vancomycin (2 g loading dose, followed by 1.75 g every 72 h) and piperacillin‐tazobactam (4.5 g loading dose, followed by 3.375 g every 8 h modified to renal function) (Day 0) was subsequently changed to ceftriaxone 2 g twice a day (Day 2) after repeat blood cultures grew *E. coli* in 4/4 bottles (time to positivity ≤ 6 h). Magnetic resonance imaging (MRI) of the spine without gadolinium (Day 1) revealed nonspecific pathology, and a repeat lumbar puncture (Day 1) was consistent with aseptic meningitis. Cerebrospinal fluid analysis demonstrated a white blood cell count of 23 cells/μL with protein of 0.7 g/L. CSF PCR testing was negative for bacterial and viral pathogens, including *Cryptococcus neoformans*, *Haemophilus influenzae*, *Neisseria meningitidis*, *Listeria monocytogenes*, and *Streptococcus pneumoniae*. Cerebrospinal fluid cultures were also negative for bacterial and viral pathogens.

**FIGURE 1:**
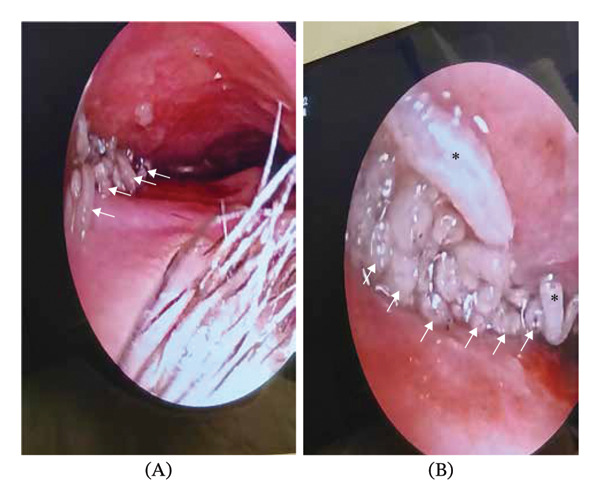
Nasal bronchoscopy showing (A) a long view of the anterior nares with multiple larvae clustered along the medial wall of the nasal vestibule (arrows) and (B) close‐up view of both crawling (asterisks) and nested (arrows) larvae.

It was during the repeat lumbar puncture that small, worm‐like organisms were observed emerging from the patient’s nose. Upon bronchoscopic examination, the organisms were identified as larvae measuring approximately 1–4 mm in length (Figure 1 and Supporting Video). Both anterior nares were infested, and there was fresh blood emerging from the underlying exposed mucosa where the larvae seemed to concentrate. Further examination of the nasopharyngeal and endotracheal airways revealed no signs of infestation of the deeper nasal cavity, nasopharynx, or endotracheal airway. No tissue biopsy, histopathologic examination, or microbiological cultures were obtained from the nasal mucosa or the larvae. A comprehensive evaluation, including bronchoscopy, otolaryngology assessment, and cross‐sectional imaging, did not reveal evidence of additional myiasis‐related lesions or alternative sites of infestation.

Samples of the larvae were collected and identified as fly maggots by the local health unit; however, the specific species could not be determined due to premature destruction of the samples. A referral to the hospital’s infectious disease team resulted in the patient receiving a single dose of ivermectin (12 mg) for a nasal myiasis diagnosis (Day 4), along with mechanical removal of the larvae using nasal irrigation and local suctioning.

MRI brain imaging and transthoracic echocardiogram (Day 2) were nondiagnostic. Patient was extubated (Day 7) without neurological deficits. Repeat MRI spine imaging with gadolinium (Day 10) demonstrated osseous edema from C4 to C6, T11/T12, L1/L2, and L5/S1, but no evidence of epidural abscess or discitis. Despite appropriate antibiotic treatment, the patient developed increasing weakness in the lower extremities (Day 15). Repeat MRI spine imaging with gadolinium (Day 15) demonstrated an extensive phlegmon extending from the skull base to the thoracic inlet measuring up to 0.8 cm thick.

The patient was transferred to a neurosurgical centre for emergent bilateral laminectomies at L4/5/S1, diskectomy at L5/S1, and drainage of the epidural phlegmon (Day 18) after developing cauda equina syndrome. Intraoperative fluid and tissue cultures were negative for bacterial growth. By the time of surgical intervention, the patient had received approximately 16 days of antimicrobial therapy, which likely contributed to the negative intraoperative cultures despite imaging and intraoperative findings consistent with infection. Strongyloides serology was positive (Day 25), but stool was negative. Follow‐up serology (Day 101) was negative. The patient completed the course of ceftriaxone on Day 49 and was discharged for rehabilitation.

## 3. Discussion

This case presents a very rare instance of nasal myiasis associated with significant morbidity, including *E. coli* bacteremia, spinal osteomyelitis, and an epidural abscess. The unique progression of this case suggests a complex interplay between myiasis and systemic infection, associated with delayed imaging findings and severe spinal complications.

### 3.1. Nasal Myiasis

**FIGURE 2:**
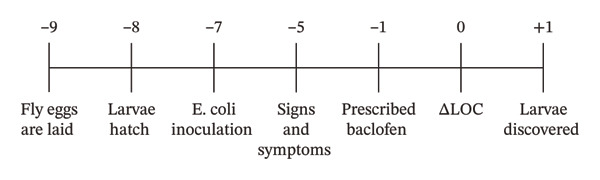
Hypothetical timeline of infection progression from egg laying to larvae discovery from time of hospital admission (Day 0). Infection progression started on Day −9 with egg laying, followed by larval hatching on Day −8 and inoculation of *E. coli* bacteria on Day −7. Signs and symptoms of metastatic infection appeared on Day −5, and on Day −1, the patient was prescribed baclofen. A change in the level of consciousness (ΔLOC) occurred on Day 0, leading to the discovery of *E. coli* bacteremia (Day 0) and larvae on Day +1.

Nasal myiasis is a rare infestation of the nasal cavity by fly larvae after deposition of their eggs by adult flies in necrotic tissues or wounds [1]. In this case, although larval specimens were initially sent for identification, definitive species confirmation could not be obtained because the specimens were destroyed and unavailable for further entomological or molecular analysis. The gross appearance and localization of the infestation, clustered in the anterior nasal vestibule with fresh mucosal ulceration and bleeding, were suggestive of a parasitic fly species capable of feeding on live mammalian tissue and potentially facilitating secondary bacterial infections. The patient’s recent travel to tropical regions further supported the likelihood of exposure to such flies, such as *Cochliomyia hominivorax* (New World screwworm fly) [1, 2]. A single adult female can lay up to 300 eggs and, after a brief incubation period of 10–20 h, the larvae hatch and begin to feed on the host’s tissue, developing over a period of 4–12 days before reaching maturity [1, 2]. Myiasis‐associated bacteremia has been described in association with organisms such as *Wohlfahrtiimonas chitiniclastica* and *Ignatzschineria indica*, which are thought to be carried by dipteran larvae [7]. A recent review further highlights the emerging zoonotic significance of these organisms in human bloodstream infections [8]. In contrast, *E*. *coli* is not among the most commonly reported pathogens directly linked to larval carriage, suggesting that in our case, bacteremia may have resulted from secondary mucosal disruption rather than primary larval‐associated transmission. Based on the incubation and development times, the patient’s *E. coli* may represent a plausible secondary invasion facilitated by mucosal disruption from nasal myiasis rather than direct larval transmission (Figure 2).

Cultures were not obtained from the larval specimens or adjacent nasal mucosa at the time of removal, as priority was given to prompt mechanical removal and treatment of the infestation. Consequently, a direct microbiological link between the nasal infestation and the *E*. *coli* bacteremia could not be definitively established, and the source of bacteremia remained presumptive, which limits confirmation of the nasal lesion as the origin of infection. Because *E*. *coli* bacteremia most commonly originates from urinary tract, hepatobiliary, or intra‐abdominal infections, these potential sources were investigated. However, CT imaging of the abdomen and pelvis demonstrated no evidence of abdominal or pelvic infection, hydronephrosis, obstructing calculi, or abscess, and no alternative infectious focus was identified.

To our knowledge, only two other cases have been reported in Canada, both infestations being nosocomially acquired [3, 4]. In the current case, the patient’s recent travel to warmer climates may have increased her exposure to flies capable of causing myiasis [2]. The patient had no underlying conditions commonly associated with myiasis, such as chronic wounds, immunosuppression, or other predisposing infections. In addition, the timeline suggests that the patient would have been relatively unaware and asymptomatic of infestation, possibly due to low larvae burden and/or altered awareness related to recent baclofen use and acute illness. In the absence of sinus opacification, necrotic black eschar, or deeper airway involvement, alternative diagnoses such as invasive fungal rhinosinusitis or mucormycosis were considered unlikely. Likewise, there were no signs of trauma, neoplasm, or necrotizing bacterial infection to account for the presence of larvae in the anterior nares.

Although *Strongyloides stercoralis* was initially considered as a possible co‐infection by the infectious diseases service, they later deemed it an incidental finding not related to nasal myiasis. *Strongyloides* spp., a parasitic nematode, typically infects the gastrointestinal tract and causes systemic symptoms through its autoinfection cycle, particularly in immunocompromised hosts [9]. While hyperinfection can be associated with Gram‐negative bacteremia, including *E. coli*, due to translocation of enteric organisms, the patient did not exhibit gastrointestinal or pulmonary manifestations suggestive of hyperinfection syndrome, and radiologic imaging along with bronchoscopic examination did not demonstrate pulmonary involvement. Furthermore, the infestation was directly visualized within the nasal cavity and appeared localized. In the absence of major immunocompromising conditions and given the lack of clinical features of hyperinfection, it is unlikely that this parasite contributed to the patient’s clinical course beyond seropositivity.

### 3.2. Altered Level of Alertness: Role of Drug Toxicity

The patient’s altered level of consciousness at presentation was likely due to prescribed medication toxicity in the presence of aseptic meningitis. The toxicology screen was positive for diltiazem, fentanyl, lidocaine, and midazolam, with the latter three presumed to be hospital‐derived. In this case, the recent ingestion of both opioids and baclofen likely contributed to central nervous system depression, leading to unresponsiveness and respiratory failure, which necessitated intubation [10, 11]. It is less likely that the aseptic meningitis aggravated her altered level of alertness given the absence of significant inflammation on the initial brain MRI. There was no evidence of trauma, seizures, or other contributing etiologies, and when she regained consciousness, she did not have any referable central nervous system deficits, suggesting a reversible etiology.

### 3.3. Imaging Findings and Sensitivity of MRI

Spinal infections, including osteomyelitis, discitis, and epidural abscesses, can be challenging to diagnose early, as imaging findings often lag behind clinical symptoms [12]. In this case, the initial MRI of the spine without gadolinium revealed subtle changes, such as increased T2 signal in the vertebral bodies and trace fluid in disc spaces, but no definitive evidence of infection. It was only later, with gadolinium‐enhanced imaging, that more pronounced findings consistent with spinal osteomyelitis and an epidural phlegmon were identified.

MRI is the imaging modality of choice for diagnosing vertebral osteomyelitis and epidural abscesses due to its high sensitivity for detecting bone marrow edema, disc space infection, and soft tissue involvement [13]. However, gadolinium contrast can significantly enhance the sensitivity of MRI by better delineating areas of inflammation and infection, particularly in the early stages of disease [13]. This case highlights the importance of serial imaging in patients with suspected spinal infections, as initial noncontrast imaging may miss early inflammatory changes.

### 3.4. Spinal Column Infections: Complications and Treatment

The progression of this patient’s spinal infection, from bacteremia to osteomyelitis, discitis, and eventually an epidural abscess with cauda equina syndrome, underscores the severe complications that can arise from untreated or inadequately treated vertebral infections [12]. Spinal epidural abscesses are associated with significant morbidity, including neurological deficits, which occurred in this patient as she developed lower extremity weakness and urinary retention [12]. Prompt surgical intervention is often required, as in this case, where the patient underwent laminectomy, diskectomy, and drainage of the epidural phlegmon.

Antibiotic therapy remains the cornerstone of treatment for spinal infections, typically requiring prolonged courses of intravenous antibiotics [14]. In this patient, ceftriaxone was continued for 6–8 weeks based on the MRI findings and recommendations by the infectious disease team. The duration of antibiotic therapy is generally guided by clinical response, repeat imaging, and the resolution of inflammatory markers [14].

## 4. Conclusion

Nasal myiasis is a rare but potentially serious condition that may be associated with systemic complications, including bacteremia and spinal infection. In this case, although a direct microbiological link could not be established, the clinical findings support a plausible association between nasal infestation and *E. coli* bacteremia in the absence of an alternative source. This case highlights the importance of early recognition, prompt management, and comprehensive evaluation of atypical sources of infection. Further reporting of similar cases is needed to better understand the mechanisms and clinical implications of myiasis‐associated bacteremia.

## Author Contributions

Faith Miller, Jesse McLean, and Giulio DiDiodato contributed equally to the design, analysis, and writing of this report.

## Funding

No funding was received for this manuscript.

## Consent

Patient informed consent was obtained and witnessed by an independent third party, and publication was approved by the institution’s Privacy Office.

## Conflicts of Interest

The authors declare no conflicts of interest.

## Supporting Information

Additional supporting information can be found online in the Supporting Information section.

## Supporting information


**Supporting Information 1** Supplemental Video: Nasal bronchoscopy video demonstrating extent of larval infestation in a single anterior nares, including fresh blood emerging from the underlying exposed mucosa.

**Supporting Information 2** CARE Checklist of information to include when writing a case report.

## Data Availability

The data that support the findings of this study are available on request from the corresponding author. The data are not publicly available due to privacy or ethical restrictions.
