# Supplementary material for: Nasal Myiasis Associated With E. coli Bacteremia, Spinal Osteomyelitis, and Epidural Abscess: A Case Report
Source: Case Rep Infect Dis. 2026 Jul 27;2026:4888769. doi: 10.1155/crdi/4888769 (PMC13403029; doi:10.1155/crdi/4888769)
Supplement: Supplementary file 2 — Supporting Information 2 CARE Checklist of information to include when writing a case report. [file CRDI-2026-4888769-s001.pdf]

CARE Checklist of information to include when writing a case report

CARE case report guidelines

(cc) BY-NC-ND

Topic	Item	Checklist item description	Reported on Line
Title	1	The diagnosis or intervention of primary focus followed by the words "case report"	1
Key Words	2	2 to 5 key words that identify diagnoses or interventions in this case report, including "case report"	44
Abstract (no references)	3a	Introduction: What is unique about this case and what does it add to the scientific literature?	23-25
	3b	Main symptoms and/or important clinical findings	27-34
	3c	The main diagnoses, therapeutic interventions, and outcomes	36-38
	3d	Conclusion—What is the main "take-away" lesson(s) from this case?	40-42
Introduction	4	One or two paragraphs summarizing why this case is unique (**may include references**)	46-56
Patient Information	5a	De-identified patient specific information.	53-54
	5b	Primary concerns and symptoms of the patient.	63-66
	5c	Medical, family, and psycho-social history including relevant genetic information	60-63
	5d	Relevant past interventions with outcomes	64-66
Clinical Findings	6	Describe significant physical examination (PE) and important clinical findings.	86-90
Timeline	7	Historical and current information from this episode of care organized as a timeline	60-119; Figure 2
Diagnostic Assessment	8a	Diagnostic testing (such as PE, laboratory testing, imaging, surveys).	68-84, 104-110
	8b	Diagnostic challenges (such as access to testing, financial, or cultural)	N/A
	8c	Diagnosis (including other diagnoses considered)	168-183
	8d	Prognosis (such as staging in oncology) where applicable	N/A
Therapeutic Intervention	9a	Types of therapeutic intervention (such as pharmacologic, surgical, preventive, self-care)	75-78; 100-102; 112-115
	9b	Administration of therapeutic intervention (such as dosage, strength, duration)	75-78; 101-102
	9c	Changes in therapeutic intervention (with rationale)	77-79
Follow-up and Outcomes	10a	Clinician and patient-assessed outcomes (if available)	104-105, 115-119
	10b	Important follow-up diagnostic and other test results	117-118
	10c	Intervention adherence and tolerability (How was this assessed?)	N/A
	10d	Adverse and unanticipated events	107-110, 113-114
Discussion	11a	A scientific discussion of the strengths AND limitations associated with this case report	151-159
	11b	Discussion of the relevant medical literature **with references**.	137-144, 161-163
	11c	The scientific rationale for any conclusions (including assessment of possible causes)	147-149
	11d	The primary "take-away" lessons of this case report (without references) in a one paragraph conclusion	229-235
Patient Perspective	12	The patient should share their perspective in one to two paragraphs on the treatment(s) they received	N/A
Informed Consent	13	Did the patient give informed consent? Please provide if requested	Yes ☑ No ☐ Via phone
